# Associations between overweight, obesity, health measures and need for recovery in office employees: a cross-sectional analysis

**DOI:** 10.1186/1471-2458-13-1207

**Published:** 2013-12-20

**Authors:** Robine E van der Starre, Jennifer K Coffeng, Ingrid JM Hendriksen, Willem van Mechelen, Cécile RL Boot

**Affiliations:** 1 Department of Public and Occupational Health, EMGO + Institute for Health and Care Research, VU University Medical Center, Amsterdam, The Netherlands; 2 Body@Work, Research Center Physical Activity, Work and Health, TNO-VUmc, Amsterdam, The Netherlands; 3 TNO (Expert Center Life Style), Leiden, The Netherlands

**Keywords:** Cross-sectional study, Need for recovery, Employees, Overweight, Obesity, Health measures

## Abstract

**Background:**

With both a high need for recovery (NFR) and overweight and obesity being a potential burden for organizations (e.g. productivity loss and sickness absence), the aim of this paper was to examine the associations between overweight and obesity and several other health measures and NFR in office workers.

**Methods:**

Baseline data of 412 office employees participating in a randomised controlled trial aimed at improving NFR in office workers were used. Associations between self-reported BMI categories (normal body weight, overweight, obesity) and several other health measures (general health, mental health, sleep quality, stress and vitality) with NFR were examined. Unadjusted and adjusted linear regression analyses were performed and adjusted for age, education and job demands. In addition, we adjusted for general health in the association between overweight and obesity and NFR.

**Results:**

A significant positive association was observed between stress and NFR (B = 18.04, 95%CI:14.53-21.56). General health, mental health, sleep quality and vitality were negatively associated with NFR (p < 0.001). Analyses also showed a significant positive association between obesity and NFR (B = 8.77, 95%CI:0.01-17.56), but not between overweight and NFR.

**Conclusions:**

The findings suggest that self-reported stress is, and obesity may be, associated with a higher NFR. Additionally, the results imply that health measures that indicate a better health are associated with a lower NFR.

**Trial registration:**

The trial is registered at the Dutch Trial Register (NTR) under trial registration number: NTR2553.

## Background

With the world rapidly urbanizing, people’s working conditions have changed significantly [1]. Figures from several countries indicate an increase in work stress and psychosocial job demands [2,3]. Over 40% of EU workers experience a high workload on a daily basis and in the Netherlands, this number even comes close to 60% [4,5]. One of the most important factors influencing an employee’s physical and mental condition is the degree to which employees are able to recover from fatigue and stress at work [6]. Need for recovery (NFR) represents the short-term effects of a day at work and is described as the need to recuperate and unwind from work-induced effort [6,7]. A high NFR can be seen as an early precursor for developing high blood pressure [8], sleeping problems [9] and fatigue [10] and is associated with subjective health complaints, future sickness absence and future cardiovascular disease [11]. Furthermore, employees with a high NFR are at an increased risk of developing occupational diseases such as burnout and musculoskeletal disorders [12].

Parallel to the increasing workload, the prevalence of overweight and obesity has increased rapidly during the last decades and has reached pandemic proportions worldwide [13,14]. In the Netherlands in 2012, estimates indicate a 31.9% and 9.8% overweight and obesity prevalence, respectively [15]. Overweight and obesity are associated with negative consequences at work, including decreased productivity, more frequent absenteeism and sick leave [16,17]. Obese employees are at a two to threefold higher risk of work disability compared to their non-obese peers [18] and obesity has been shown to be a predictor of long-term sick leave [19]. If obesity levels remain unchanged, the growing obesity-related health burden will have large economic consequences. These will include an increase in costs [20,21], both direct (as overweight and obesity are major risk factors for certain chronic diseases, including cardiovascular diseases and different forms of cancer [18,22,23]) and indirect (costs related to absenteeism, loss of productivity and disability) [16,17].

Following this line, both a high NFR and overweight and obesity are a potential burden for organizations (e.g. productivity loss and sickness absence) [11,18]. However, no studies have been conducted to test whether there is an association between these two variables. Furthermore, as overweight and obese persons tend to report a poorer health than their normal body weight peers [24-26], it is of added value to examine the association between health measures and NFR. To the best of our knowledge, health measures and their association with NFR have not been investigated so far. Therefore, it is interesting to examine the association between subjective health measures, such as general health, mental health, sleep quality, stress and vitality, and NFR.

The aim of this paper is twofold; 1) to examine the association between overweight and obesity and NFR and, 2) to examine the association between health measures (general health, mental health, sleep quality, stress and vitality) and NFR. We hypothesize that both overweight and obesity are associated with a higher NFR compared to normal body weight and that health measures that indicate a better health will be associated with a lower NFR.

## Methods

### Study sample & recruitment

In this study, data from the baseline measurement of the Be Active & Relax “Vitality in Practice” (VIP) project were used [27]. This randomized controlled trial was set up to evaluate a physical activity and relaxation intervention aimed at improving NFR in office workers. In September 2011, all 1,182 office employees (> 18 years) of a single financial service provider received an invitation to participate in the project. Those on sick leave for more than four weeks were not eligible to participate. An off-line questionnaire was administered at baseline, including measures of NFR, daily physical activity, general health, mental health, sleep quality, stress and vitality. All data were self-reported. A total of 414 employees (response rate: 35%) from 19 departments signed the informed consent form, after which 412 employees completed the baseline questionnaire (providing data on NFR) and were included in the Be Active & Relax project. This study was approved by the Medical Ethics Committee of the VU University Medical Center Amsterdam. The development and design of the Be Active & Relax project has been described in full detail elsewhere [27].

### Dependent variable

In this study, ***need for recovery (NFR)*** was assessed using the Need for Recovery after Work scale of the Dutch version of the Questionnaire of the Experience and Evaluation of Work (Dutch abbreviation: VBBA). The used subscale of the VBBA, a questionnaire on the perception and judgement of work [28], consists of 11 dichotomous items (yes/no). These items represent short-term effects of a day at work, with questions like “I find it hard to relax at the end of a working day” and “When I get home, people should leave me alone for some time”, which were coded ‘0’ or ‘1’ in such a way that higher scores are related to more complaints. The NFR scale was computed by summing up the scores of the 11 items, of those providing data for at least 8 of the 11 items. The NFR total score was standardised to a score ranging from 0–100, based on the number of items with valid data. When three items are missing, the total score is expressed as percentage of the eight items, instead of the eleven items (the number of scored points was divided by the number of answered items and multiplied by 100). Higher scores indicate a higher NFR after work. Internal consistency of the scale was tested in this study and was found to be of good quality (α = 0.85), which is comparable to previous studies on NFR [28,29]. Employees with percentage scores of 54.5 or higher (a cut-off point of six or more positive responses, as recommended by Broersen *et al*.) [30,31] were considered to have a high NFR, as previous research showed that they have a higher risk for developing psychological complaints than people with a percentage score below 54.5 [28,30,32].

### Independent variables

***Overweight and obesity*** were investigated by calculating BMI as the body weight in Health Organization recommendations [33]. Body weight and body height were assessed by self-report. BMI was categorized into three categories: normal body weight (cut-off point: <25 kg/m^2^), overweight (cut-off point: 25- < 30 kg/m^2^), and obesity (cut-off point: ≥30 kg/m^2^) [33]. Those participants (1.9%) categorized as underweight (<18.5 kg/m^2^) were included in the normal body weight category because the number of participants did not allow a separate category (n = 6). For the same reason, participants categorized as extremely obese (≥ 35 kg/m^2^, n = 9), were included in the overweight category. Normal body weight was chosen as reference category in the analyses.

### Health measures

The following health measures were investigated: general health, mental health, stress, sleep quality and vitality.

***General health and mental health*** were measured by items of the Dutch validated version of the Rand-36 measure of health-related quality of life [34]. General health perceptions were measured by asking employees to give an indication on how they perceived their health on a 5-pointscale (bad, moderate, good, very good or excellent) and to indicate on four propositions (e.g. “I more easily fall ill than others”) to which extent on a 5-pointscale they “totally agreed” or “totally disagreed”. To assess mental health, employees were asked to indicate how often they had felt nervous, down, calm/relaxed, depressed/gloomy and tired, during the past four weeks. In this study, the Rand-36 measure of health-related quality of life has shown satisfactory internal consistency (α = 0.85) for the assessment of mental health and reasonable internal consistency (α = 0.73) for the assessment of general health. The internal consistency found in this study for mental health is comparable to other studies and for general health is slightly lower than previous research [34]. Both the general and mental health scale were computed by summing up the scores of the 5 items (in those providing data for at least 3 of the 5 items). The general and mental health total scores are transformed to a 0–100 range (as percentage of maximum total score) and all items are averaged in the same scale together. When items are missing, the scale average is filled, assuming that the respondent would have answered this item in a similar way as the others. Certain items were transformed such that higher scores indicate a better health status.

To measure ***stress***, the Dutch short form of the Perceived Stress Scale (PSS) was used [35]. Participants were asked to indicate on a 5-pointscale (“never” to “very often”) how often they had had certain feelings during the last month (e.g. “In the last month, how often have you felt that you were unable to control the important things in your life?”). Items were coded in such a way that higher scores indicate a higher level of stress. PSS-4 is considered to be sound, but previous studies have found a rather low internal reliability (α = 0.60) [35]. In this study, however, a satisfactory internal consistency was found (α = 0.70).

***Sleep quality*** was assessed by the Dutch Jenkins Sleep Problems Scale [36]. This scale contains four items, i.e., trouble falling asleep, trouble to continue sleeping, waking up feeling tired and worn out, and trouble staying awake during the day. Participants were asked to indicate how often they had experienced the four criteria mentioned above in the past month (‘0 days’ to’22-31 days’), coded in such a way that higher scores indicate a better sleep quality. In this study, a marginal satisfactory Cronbach’s alpha value of 0.61 was found, which is comparable to previously found internal consistency for the same construct [36].

***Vitality*** was assessed using a part of the Utrecht Work Engagement Scale (UWES) [37], which contains 6 items (e.g. “At my work, I feel myself bursting with energy”) that had to be answered on a 7-pointscale (“never” to “always”). Answers were coded in a way that higher scores designate better vitality. In this study, vitality indicates a satisfactory internal consistency (α = 0.82). Previous studies reported comparable or even lower internal consistency (α = 0.68-0.80) [37,38].

### Potential confounders

***Sociodemographic variables***, such as gender, marital status (married/cohabitating, in a relation, no cohabitating, single, divorced, widowed) and educational level were self-reported. Educational level was divided into lower education (no education, primary school, lower vocational education or lower secondary school), middle education (intermediate vocational education or intermediate/higher secondary education) and higher education (higher vocational education and university). Although all from one single service provider, a great diversity in job types was found among respondents. As the job types within the financial service provider are related to educational level, educational level was examined as a potential confounder as a proxy for job type/skill level. Age was calculated by extracting the self-reported date of birth from the date of completion of the questionnaire by participant.

***Job demands*** were taken into account, as previous studies have identified job demands to be associated with NFR [39]. This work-related variable was assessed using the items “work fast”, “work hard”, “no excessive work”, “enough time” and “conflicting demands” [40] (4-point scale from “strongly agree” to “strongly disagree”) which are part of the validated Dutch version of the Job Content Questionnaire (JCQ). An acceptable Cronbach’s alpha value was found in this study for job demands (α = 0.79). This is consistent with previous studies in different countries, including the Netherlands [40].

***General health***, described previously, was included as potential confounder in the relationship between overweight and obesity and NFR. Literature shows that obesity and general health are related [41-43]. Obese persons, as compared to their normal weight peers, seem to be more likely to have adverse health outcomes, such as poor general health [41,43]. As general health and overweight/obesity seem to be associated, we aimed at investigating the independent association between overweight and obesity and NFR, by correcting for associations between general health and NFR.

### Statistical procedures

Descriptive analyses were performed to summarize the characteristics of the population using means and standard deviations or percentages. For the outcome variable (NFR), a square root transformation was formally applied as its distribution was positively skewed, due to the high number of respondents scoring the minimum score of NFR 0–20 (37.7%). This percentage is consistent with other studies on NFR [12]. However, the square root transformation did not meaningfully improve the distribution and therefore no transformation of original values was applied, which is in line with research of de Croon *et al.*[12]. Univariate linear regression analyses were used to determine the associations with NFR, which was treated as a continuous outcome variable, and each independent variable.

Potential confounders were included in the adjusted analyses as confounders when the Beta coefficient of the independent variable changed at least 10% following addition of the potential confounder to the model. Furthermore, potential effect modification by age, gender and job demands was tested in the adjusted models. For each effect modifier, a linear regression model was fitted by crossing a predictor (overweight, obesity and health measures) and a modifier and adding this interaction term to the regression model. Thereby, the interaction between overweight/obesity and general health was examined by performing an analysis of variance (two-way ANOVA F-test). The level of significance was set at p < 0.05. Data were analysed using SPSS Version 20.0 (SPSS Inc., Chicago, IL, USA).

## Results

### Study population

The participating employees were on average 41.3 years old (SD = 10.3), ranging from 19 to 63 years. Average BMI was 24.9 (SD = 4.0) and most employees (58.5%) were classified as having a normal body weight (BMI < 25 kg/m^2^), 31% was classified as overweight and 10.5% was classified as obese. For two participants, BMI could not be calculated because of missing data on body weight and/or height. The overall mean score of NFR in the total study population was 32.2 (SD = 29.3) and a total of 22.8% showed a high need for recovery (>54.5). The mean NFR for the normal body weight, overweight and obesity group were 32.4 (SD = 27.6), 27.3 (SD = 29.2) and 45.9 (SD = 34.9) respectively. The population characteristics are summarized in Table 1.

**Table 1:** Characteristics of the study population

**Characteristics**	**n (%)**	**Mean (SD)**
Age (years) (n = 413)		41.3 (10.3)
Female gender (n = 414)	164 (39.6)	
Dutch ethnicity (n = 413)	372 (89.9)	
BMI (n = 410)		24.9 (4.0)
Normal body weight (<25 kg/m^2^)	240 (58.5)	
Overweight (25–29.9 kg/m^2^)	127 (31.0)	
Obese (≥30 kg/m^2^)	43 (10.5)	
College/university education (n = 413)	233 (56.4)	
Married/cohabitating (n = 414)	312 (75.3)	
Need for recovery (NFR) (n = 412)		32.2 (29.3)
Low (<=54.5)	318 (77.2)	
High (> 54.5)	94 (22.8)	

BMI; Body Mass Index, n; number of cases, SD; standard deviation.

### Overweight and obesity & health measures

Results of the unadjusted and adjusted linear regression models are shown in Table 2. Unadjusted analyses showed significant associations for all health measures (general health, mental health, sleep quality, stress and vitality) and obesity with NFR.

**Table 2:** Unadjusted and adjusted linear regression models on need for recovery

	**Need for recovery**					
**Exposure variable**	**Unadjusted analysis**			**Adjusted analysis**^**A**^		
	**β**	**(95%CI)**	**p-value**	**β**	**(95%CI)**	**p-value**
**BMI categories**						
Normal body weight (<25 kg/m^2^)	reference		reference		reference	
Overweight (25–29.9 kg/m^2^)	−5.15	(−11.40, 1.10)	0.106	−3.55^B^	(−9.43, 2.34)	0.237
Obesity (≥ 30 kg/m^2^)	13.45	(4.03, 22.88)	0.005	8.77^B^	(0.01, 17.56)	0.050
**Health measures**						
General health	−0.61	(−0.78, -0.46)	<0.001	−0.58	(−0.73, -0.44)	<0.001
Mental health	−1.03	(−1.19, -0.87)	<0.001	−0.93	(−1.09, -0.76)	<0.001
Sleep quality	−16.15	(−18.75, -13.56)	<0.001	−14.06^C^	(−16.60, -11.51)	<0.001
Stress	20.26	(16.72, 23.80)	<0.001	18.04	(14.53, 21.56)	<0.001
Vitality	−10.84	(−13.73, -7.96)	<0.001	−10.58^C^	(−13.29, -7.89)	<0.001

BMI; Body Mass Index, ^A^Adjusted for age, education and job demands, ^B^Additionally adjusted for general health, ^C^Adjusted for effect modification by job demands.

Adjusted analyses also showed significant association between health measures and NFR; significant positive associations (p < 0.001) were observed between general health, mental health, sleep quality and vitality, and NFR. These results suggest that a better general health, better mental health, better sleep quality and a better vitality are all associated with a lower NFR. On the contrary, the significant negative association (p < 0.001) between stress and NFR suggests that self-reported stress is associated with a higher NFR. After adjustment for confounding by age, educational level, job demands and general health, the significant positive association between obesity and NFR observed in the unadjusted analysis (B = 13.45, 95%CI:4.03, 22.88, p = 0.005) changed into a borderline significant positive association (B = 8.77, 95% CI:0.01, 17.56, p = 0.05), with normal body weight as the reference category. This finding points in the direction that obesity as compared to normal body weight may be associated with a higher NFR. No strong evidence was found to support an association between overweight and NFR, compared to normal weight. A significant interaction in the adjusted model was identified for job demands in the association between NFR and sleep quality (B = −14.06, 95% CI: -16.60, -11.51) and vitality (B = −10.58, 95% CI: -13.29, -7.89). Subgroup analyses revealed different associations between sleep quality, vitality and NFR in employees with high job demands and employees with low job demands, with the strongest significant negative association found in employees with high job demands. No effect modification for age and gender was observed and no significant interaction between overweight/obesity and general health was found.

## Discussion

The purpose of this study was to examine the association between overweight and obesity, health measures and need for recovery (NFR) in Dutch office employees. Our results showed that obesity and poor health status were associated with NFR, indicating that obesity and poor general health, poor mental health, poor sleep quality, self-reported high stress levels and poor vitality were associated with a high NFR. No significant associations were found between overweight and NFR in either unadjusted or adjusted models. After additionally adjusting for general health in the association between obesity and NFR, the positive association between obesity and NFR remained significant, although the association observed was attenuated. This indicates that obesity is independently associated with NFR, but that obesity is also partly associated with NFR as a result of poor underlying general health. Given the substantial reduction of the strength of the association after adjustment for confounding, this result has to be interpreted with caution.

Previous studies showed that both overweight and a high NFR are important factors in developing long-term health problems, sickness absence and productivity loss [11,16,17]. To date, there is a lack of evidence on the associations between overweight and obesity and NFR. In this study it was found that obesity, as compared to normal body weight, was associated with a high NFR. Since obesity and NFR are associated with subjective health complaints, future sickness absence and occupational diseases such as burnout and musculoskeletal disorders [11,12,16,17,19], it is important to further explore this association and to determine its causality.

In contrast to our expectations, we did not find a significant association between overweight and a high NFR. A negative tendency – although non-significant – was observed, which suggests that overweight, as compared to normal body weight, is associated with a lower NFR in office employees. This opposite direction is in accordance with the results of a recently performed systematic review, which found that overweight, as compared to normal weight, was associated with a significantly lower all-cause mortality whereas obesity was associated with a significantly higher all-cause mortality [44]. A possible explanation for the difference in direction of the associations found can be the large number of employees with a BMI slightly above the 24.9 kg/m^2^, which classifies them in the overweight instead of normal body weight group. This may underestimate the association between overweight and NFR.

As overweight and obese persons tend to report a poorer self-rated health than their normal body weight peers [24-26], the association between other health measures and NFR are also important to examine in addition. In this study, a significant positive association between stress and NFR was found, suggesting that high levels of self-reported stress are associated with a higher NFR. A limited number of studies examined incomplete recovery in association with health complaints and stress. These few studies found similar results, as they showed that poor recovery after work was significantly associated with worse health status [9,12] and long-term stress [45]. Furthermore, significant negative associations were observed in this study between the remaining health measures and NFR, suggesting that a better general health, better mental health, better sleep quality and better vitality are associated with a lower NFR. The associations observed here are a novel finding, as previous studies did not examine associations between these health measures and NFR.

### Strengths and limitations

To our knowledge, this is the first study in which the association between overweight and obesity and NFR was studied. Thereby, this is one of the first studies with a focus on the association between health measures and NFR.

A major strength of the present study is the large sample size, which provided sufficient statistical power to examine associations between overweight, obesity, health measures and NFR in office employees. Thereby, the response rate (35%) was acceptable for studies within worksites. A meta-analysis (61 studies published in 2000 and 56 studies in 2005) showed that this percentage is consistent for studies within worksites [46].

In addition, the prevalence of normal body weight (including underweight), overweight and obesity are comparable to the prevalence in the total Dutch population (58.5% classified as normal weight, 31% classified as overweight, 10.5% classified as obese in our study versus 58.3% normal weight, 31.9% overweight, 9.8% obese in the total Dutch population) [15], which makes our study population a representative population. However, when interpreting the results it is important to keep in mind that this study was performed among sedentary office employees. Thereby, those on sick leave for more than four weeks were not eligible to participate and therefore our findings are not generalizable to populations with a high absenteeism rate.

A major limitation of this study is its cross-sectional design. Within this design, no conclusions regarding the causal directions of the associations can be drawn. This implies that NFR could either be increased due to obesity, or that a high NFR could contribute to the development of obesity (i.e. too tired to prepare healthy meals and/or engage in regular physical activity). In addition, the question remains whether a high NFR after work is an antecedent or a consequence of poor health. Another issue concerns a possible misclassification of the measurement of overweight and obesity. The estimation of overweight and obesity in this population is not perfect as it is solely based on self-reported body height and body weight. Self-reports of weight and height produce under-estimates as both men and women tend to overestimate their height and underestimate their weight [18,47]. Nevertheless, several studies, among others a study in a Dutch overweight working population, showed that self-reported body weight and height are of satisfactory reliability for the assessment of overweight and obesity [48] and are valid for identifying relationships in epidemiological studies [19]. Additionally, all information on independent and dependent variables was obtained using self-reports. Even if the questionnaire was designed to minimize self-report bias in responses, some items may have been subject to this type of bias. Finally, as the aim of this study was exploratory, we did not correct our p-values for multiple testing by e.g., the Bonferroni correction [49]. As the p-values of the results we found were at least ten times smaller than 0.05, applying the Bonferroni correction would not have changed our results.

### Implications for practice and research

Given that recovery from work is important to maintain good health and well-being [50], strategies to reduce the need for recovery are important. It is important to identify factors that are associated with a high NFR, as well as the causal relationship between associated variables. Understanding the associations may contribute to understanding, and eventually reversing high NFR among office employees. These findings can support in the development of intervention strategies by addressing the unique concerns of workers and aspects of the workplace. The associations observed in this study are an important concern also for public health policy, as overweight, obesity, poor health status and NFR have considerable implications for morbidity [11,18]. A next step is to examine the causality of the associations observed here to develop prevention and intervention measures, in order to reduce the NFR among office employees. Follow-up studies are also needed to explore eating behaviour and lifestyle-related factors which are probably important mediating factors in the relationship between overweight, obesity and a high NFR.

## Conclusion

Our findings confirmed our hypotheses that obesity is associated with a high NFR and that good health is associated with a low NFR. The results should be interpreted with caution. Because of its cross-sectional design, no causal relations can be identified. Given that recovery from work is important to protect employees’ health and well-being, policies to decrease the NFR are important. As our findings have potentially important health implications, it is urgent to further explore the causal relationships involved in the associations observed here. Future studies should therefore focus on these outcomes and examine the effects of obesity and health measures on NFR in intervention trials.

## Abbreviations

NFR: Need for recovery; BMI: Body mass index.

## Competing interests

The authors declare that they have no competing interests.

## Authors’ contributions

REvdS wrote the first draft of the manuscript. JKC, IJMH, WvM and CRLB provided intellectual input and had a role in supervision. All authors have read and approved the final manuscript.

## Pre-publication history

The pre-publication history for this paper can be accessed here:

http://www.biomedcentral.com/1471-2458/13/1207/prepub
